# Diagnostic accuracy and clinical utility of mTLICS versus TLICS and TL AOSIS in stratifying three-tier treatment for thoracolumbar injuries: focus on intermediate score range

**DOI:** 10.1186/s12891-025-09124-7

**Published:** 2025-09-01

**Authors:** Quang Anh Dao, Van Son Nguyen, Van Quang Dang, Phuong Chinh Tran, Dinh Thanh Son Le

**Affiliations:** 1 Department of Diagnostic Imaging, Phu Tho Provincial General Hospital, Phu Tho, Vietnam; 2 Department of Neurosurgery, Phu Tho Provincial General Hospital, Phu Tho, Vietnam; 3 Outpatient Department, Phu Tho Provincial Obstetrics and Pediatrics Hospital, Phu Tho, Vietnam; 4 Department of Urology, Phu Tho Provincial General Hospital, Phu Tho, Vietnam

**Keywords:** Modified TLICS, Thoracolumbar spine injury, Magnetic resonance imaging, Posterior ligamentous complex, Treatment decision-making, Minimally invasive surgery, Surgical management, ROC analysis, Decision curve analysis

## Abstract

**Background:**

Thoracolumbar injury classification systems such as TLICS and TL AOSIS are widely implemented but offer limited guidance in intermediate score ranges (TLICS = 3–4), where treatment decisions are often uncertain. The modified TLICS (mTLICS) was developed to address this gap by integrating MRI-derived quantitative parameters.

**Methods:**

This retrospective study included 146 adults with MRI-confirmed thoracolumbar spine injuries (T1–L5) treated at Phu Tho Provincial General Hospital between April 2024 and May 2025. Inclusion required MRI within 7 days of trauma and complete clinical data, including ASIA grade, VAS score, and treatment modality. All cases were classified using TLICS, TL AOSIS, and mTLICS, and managed conservatively, minimally invasively, or surgically. Predictive performance for treatment allocation was assessed using ROC analysis, multinomial logistic regression, and decision curve analysis (DCA).

**Results:**

mTLICS showed the highest diagnostic accuracy across all treatment comparisons (AUC = 0.94-1.00), particularly in the intermediate-score group (TLICS = 3–4), with AUCs of 0.991 (conservative vs. surgical) and 0.965 (minimally invasive vs. surgical). Multinomial regression identified mTLICS as the sole independent predictor of treatment allocation (OR = 31.2-1338.4; *p* < 0.01), while TLICS and TL AOSIS were not statistically significant. DCA demonstrated the highest net clinical benefit for mTLICS, especially within the 0.3–0.6 threshold range.

**Conclusions:**

The mTLICS demonstrated improved accuracy in stratifying thoracolumbar injuries across three treatment tiers and enhanced clarity in decision-making for intermediate cases. Its MRI-based components support personalized, image-guided management. Nonetheless, as clinicians at the study site were familiar with the mTLICS framework through prior academic exposure, potential incorporation bias cannot be entirely excluded. These findings should therefore be interpreted with caution, as mTLICS scores were calculated retrospectively after treatment completion and were not used prospectively to determine patient management, underscoring the need for prospective, multicenter validation to confirm its generalizability.

## Introduction

Thoracolumbar spine injury (T1–L5) is one of the most prevalent and clinically significant forms of spinal trauma, particularly in regions with high incidences of falls and road traffic accidents. According to the WFNS Spine Committee, its global incidence exceeds 30 cases per 100,000 annually and continues to rise in tandem with aging populations and increased occupational hazards [1]. The thoracolumbar junction (T11–L2), a biomechanical transition between the rigid thoracic spine and the more mobile lumbar region, is especially susceptible to instability [2]. To aid treatment decision-making, classification systems such as the Thoracolumbar Injury Classification and Severity Score (TLICS) [3] and the Thoracolumbar AO Spine Injury Score (TL AOSIS) [4] are commonly employed. While the AO system provides a widely accepted morphological framework, it does not independently guide management. TL AOSIS addresses this gap by incorporating neurological status and clinical modifiers into a numeric score, thereby facilitating treatment stratification similar to TLICS. However, both systems lack integration of key imaging-based markers of instability, including posterior ligamentous complex (PLC) disruption, vertebral body height loss, and canal compromise, which are strongly associated with adverse outcomes [5–7]. In response to these limitations, An et al. questioned the added value of TL AOSIS over TLICS and advocated for imaging-informed refinements [7].

Magnetic resonance imaging (MRI) has become the gold standard for identifying instability in thoracolumbar trauma due to its superior sensitivity compared with CT and radiographs, especially in neurologically intact patients [8, 9]. To improve consistency, Aly et al. proposed a standardized MRI-based classification algorithm [10]. Nevertheless, existing systems were originally designed to guide binary treatment decisions between conservative and surgical management. They do not adequately account for the growing use of minimally invasive surgery (MIS), including vertebroplasty and percutaneous instrumentation, which has gained acceptance in selected cases [11, 12]. These intermediate interventions are increasingly used in neurologically intact patients with MRI-detected instability, such as burst fractures, ≥ 50% vertebral height loss, or PLC disruption, regardless of bone mineral status [5, 11, 12]. MIS therefore represents a separate therapeutic tier that occupies a position between conservative and open surgical care. Although Park’s modified TLICS (mTLICS) was developed to improve stratification between conservative and surgical approaches, it did not formally define MIS as an independent treatment category [5]. Our study expands upon this framework by operationalizing MIS as a third therapeutic tier, guided by MRI-based indicators of mechanical instability. This adjustment aligns with contemporary clinical practice, as supported by Gao and Song, who demonstrated the effectiveness of MIS across both osteoporotic and non-osteoporotic populations [11, 12]. Consequently, we adopt a three-tier treatment model comprising conservative, MIS, and surgical management to reflect real-world decision-making more accurately and to evaluate classification system performance in a comprehensive manner. This approach is consistent with recent literature calling for imaging-guided, individualized scoring frameworks [5, 11].

A persistent challenge in using conventional scoring systems lies in the intermediate range of TLICS scores (3–4), where treatment recommendations often lack clarity. Studies by Lucasti, Smith, and Khil reported frequent surgical interventions among patients initially classified for conservative care [13–15]. To address such discrepancies, Park introduced mTLICS, which was subsequently validated by Withrow et al. [5, 6]. This enhanced system integrates three quantitative MRI parameters, namely graded PLC injury, ≥ 50% vertebral height loss, and ≥ 50% canal compromise, in combination with ASIA neurological grading. Early validation studies demonstrated that mTLICS provides superior predictive accuracy and greater concordance with actual clinical decisions compared to TLICS and TL AOSIS [6].

Despite these advantages, the utility of mTLICS in supporting three-tier treatment stratification, including MIS, has not yet been comprehensively assessed. Recent contributions, including Park’s framework and Gao’s treatment-focused review, highlight the growing need for radiologically driven, individualized models that incorporate both anatomical severity and neurological status to guide management [5, 11]. These developments establish a strong conceptual rationale for implementing evidence-based, tiered treatment algorithms in thoracolumbar trauma care.

Accordingly, this study aims to evaluate and compare the performance of mTLICS, TLICS, and TL AOSIS in stratifying thoracolumbar spine injuries into three clinically relevant treatment categories: conservative, minimally invasive, and surgical. Particular attention is given to the intermediate-score subgroup (TLICS 3–4), where traditional systems often yield ambiguous guidance. The overarching goal is to validate mTLICS as a reliable, MRI-guided decision-support tool to facilitate individualized and stratified management of thoracolumbar spine injuries.

## Methods

### Study design and setting

This retrospective cohort study was conducted at Phu Tho Provincial General Hospital, a provincial tertiary care centre in northern Vietnam, between April 2024 and May 2025. The study aimed to evaluate the prognostic performance of the mTLICS classification compared to the conventional TLICS and TL AOSIS systems in stratifying thoracolumbar spine injury patients into three treatment strategies: conservative, minimally invasive, and surgical. The study followed the STROBE reporting guidelines and received institutional ethics approval (Approval No. 03/TB-HĐĐĐ, April 18, 2025). Although clinicians at our institution were acquainted with the mTLICS framework through prior academic exposure, all treatment decisions during the study period were made by multidisciplinary consensus in accordance with institutional protocols and established guidelines (TLICS, TL AOSIS, WFNS Spine Committee). The mTLICS scores were calculated retrospectively from anonymized imaging and clinical data after treatment completion and were not used prospectively to determine patient management.

### Patient population

Eligible participants were adults (≥ 18 years) with traumatic thoracolumbar spine injuries (T1–L5), confirmed by clinical assessment and imaging. Inclusion required MRI within 7 days of injury, with complete sequences including sagittal T1-weighted, T2-weighted, STIR or Dixon, and axial T2-weighted images. Only patients with full clinical documentation were included, comprising VAS pain scores, ASIA neurological grading (AIS A–E), and a clearly recorded treatment plan.

At our institution, MRI is routinely performed in patients who are neurologically intact, show inconclusive findings on radiography or CT, or are being evaluated for conservative or minimally invasive treatment. This approach reflects growing consensus on MRI’s superior sensitivity in detecting PLC disruption, canal compromise, and subtle instability, especially in cases with TLICS scores of 3 to 4 where treatment decisions are less straightforward. Accordingly, MRI served as the standard imaging modality to ensure consistent classification and treatment allocation.

Of 168 patients initially screened, 22 were excluded due to incomplete clinical records (*n* = 11), MRI performed more than 7 days post-injury (*n* = 7), or poor image quality (*n* = 4). Ultimately, 146 patients met all inclusion criteria and were analyzed.

**Fig. 1:**
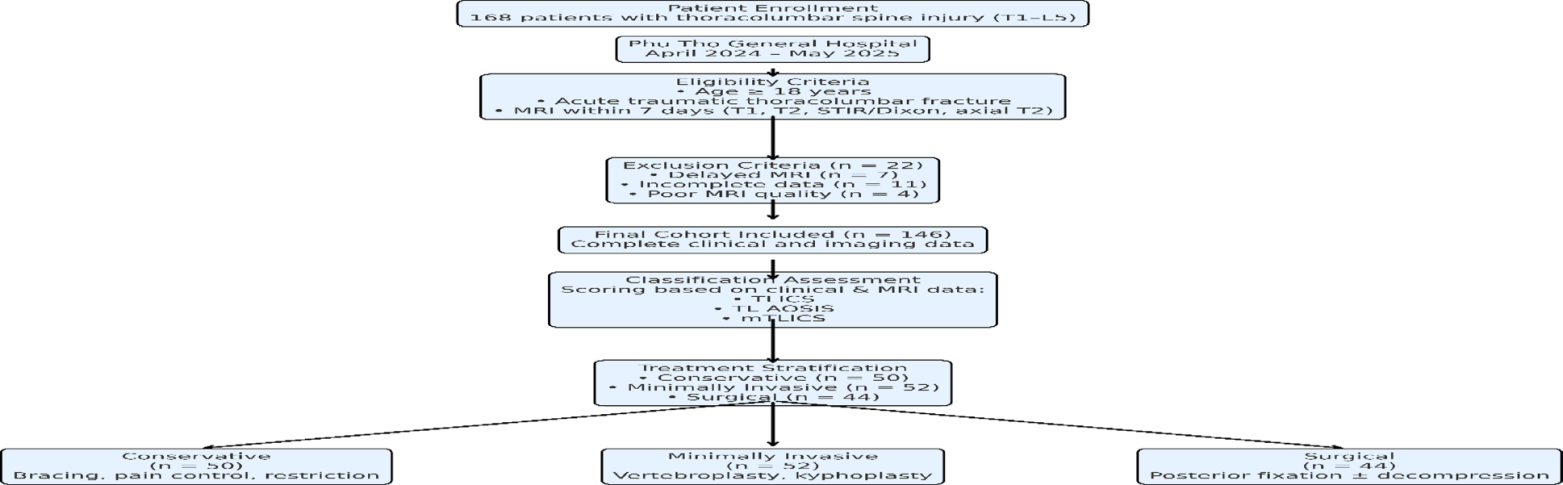
Study flowchart illustrating patient eligibility, exclusion criteria, classification assessment, and treatment stratification

**The patient selection process is illustrated in** Fig. 1.

### Data collection, imaging assessment, classification, and treatment stratification

Demographic, clinical, and imaging data were retrieved retrospectively from hospital electronic records. The demographic parameters collected included age, sex, and occupational status. Clinical variables included mechanism of injury, which was categorized into traffic accidents, occupational trauma, or domestic falls. Neurological status at admission was assessed using the ASIA Impairment Scale (AIS A–E), while pain intensity was documented using the Visual Analog Scale (VAS).

All patients underwent spinal MRI on a 3.0 Tesla scanner (Siemens, Germany) using a standardized trauma protocol. Sequences included sagittal T1-weighted, T2-weighted, STIR or Dixon, and axial T2-weighted images, based on previously validated MRI frameworks for thoracolumbar trauma evaluation [5, 8, 9]. All MRI scans were independently reviewed by a board-certified diagnostic radiologist with over five years of dedicated experience in spinal trauma interpretation. Radiological features assessed included fracture morphology (compression, burst, distraction, or translation) [3], vertebral body height loss (dichotomized as < 50% or ≥ 50%), and spinal canal compromise (defined as anterior-posterior narrowing ≥ 50%) [5, 6].

Posterior ligamentous complex (PLC) integrity was evaluated on STIR or Dixon sequences and graded on a four-point MRI-based scale (0 = intact, 1 = Focal edema in the soft tissue of PLC on MRI, 2 = Focal edema in the bony structure of the facet joint or spinous process, 3 = Definite discontinuation of the PLC) as proposed by Park [5] and later validated by Mehta [8]. For analysis, PLC injury was dichotomized as present (≥ 1) or absent (0). In cases of diagnostic uncertainty, MRI interpretations were collaboratively reviewed by the diagnostic radiologist and spine surgical team, in line with institutional protocols.

Patients were classified using three established systems. Specifically, the TLICS system incorporated morphology, PLC status, and neurological findings [3]. The TL AOSIS included injury type (A–C), neurologic modifiers (N0–N4), and clinical modifiers (M1: osteoporosis; M2: polytrauma/comorbidities) [4]. The mTLICS system extended TLICS by integrating quantitative MRI findings, including PLC injury grading, vertebral body height loss ≥ 50%, and canal compromise ≥ 50%, as recently validated in treatment prediction models [5, 6].

To illustrate the real-world applicability of these classification systems, for representative clinical cases are presented below.

**Fig. 2:**
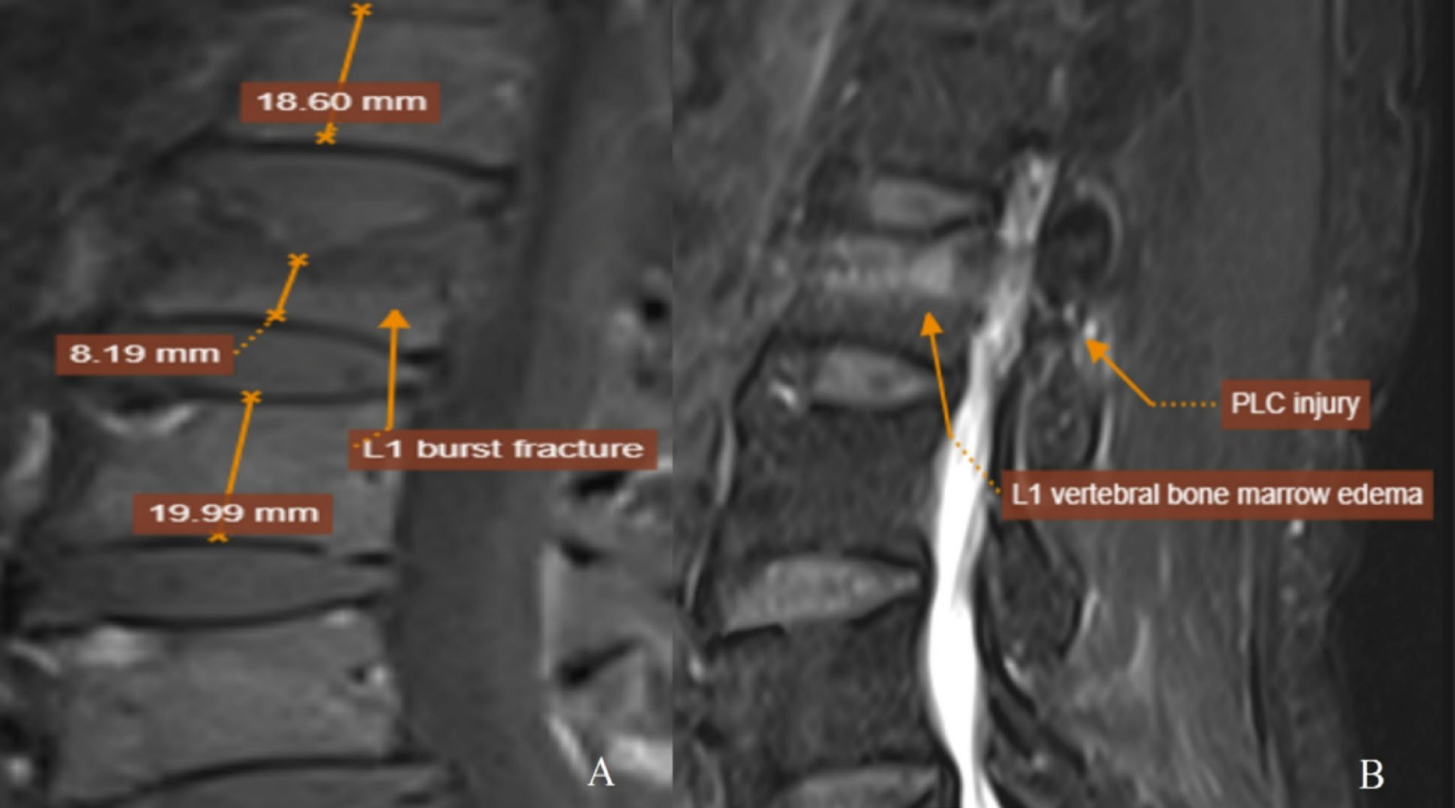
High-resolution MRI illustration (T1W, T2W-Dixon) of a patient with thoracolumbar burst fracture. MRI showing L1 burst fracture with > 50% height loss (**A**) and PLC injury (**B**); mTLICS = 5. Treated with posterior screw fixation (Images exported in 300 dpi directly from PACS)

**The first case is shown in** Fig. 2.

A female patient in her 60s was admitted following a motor vehicle accident. MRI revealed a burst fracture of the L1 vertebral body with > 50% height loss (A) and high signal intensity in the posterior ligamentous complex on Dixon imaging, indicating ligamentous injury (B). The injury was scored as TL AOSIS = 4 (morphology: 3, PLC: 1), TLICS = 4 (morphology: 2, PLC: 2), and mTLICS = 5 (morphology: 3, PLC: 2). The patient underwent posterior pedicle screw fixation.

**Fig. 3:**
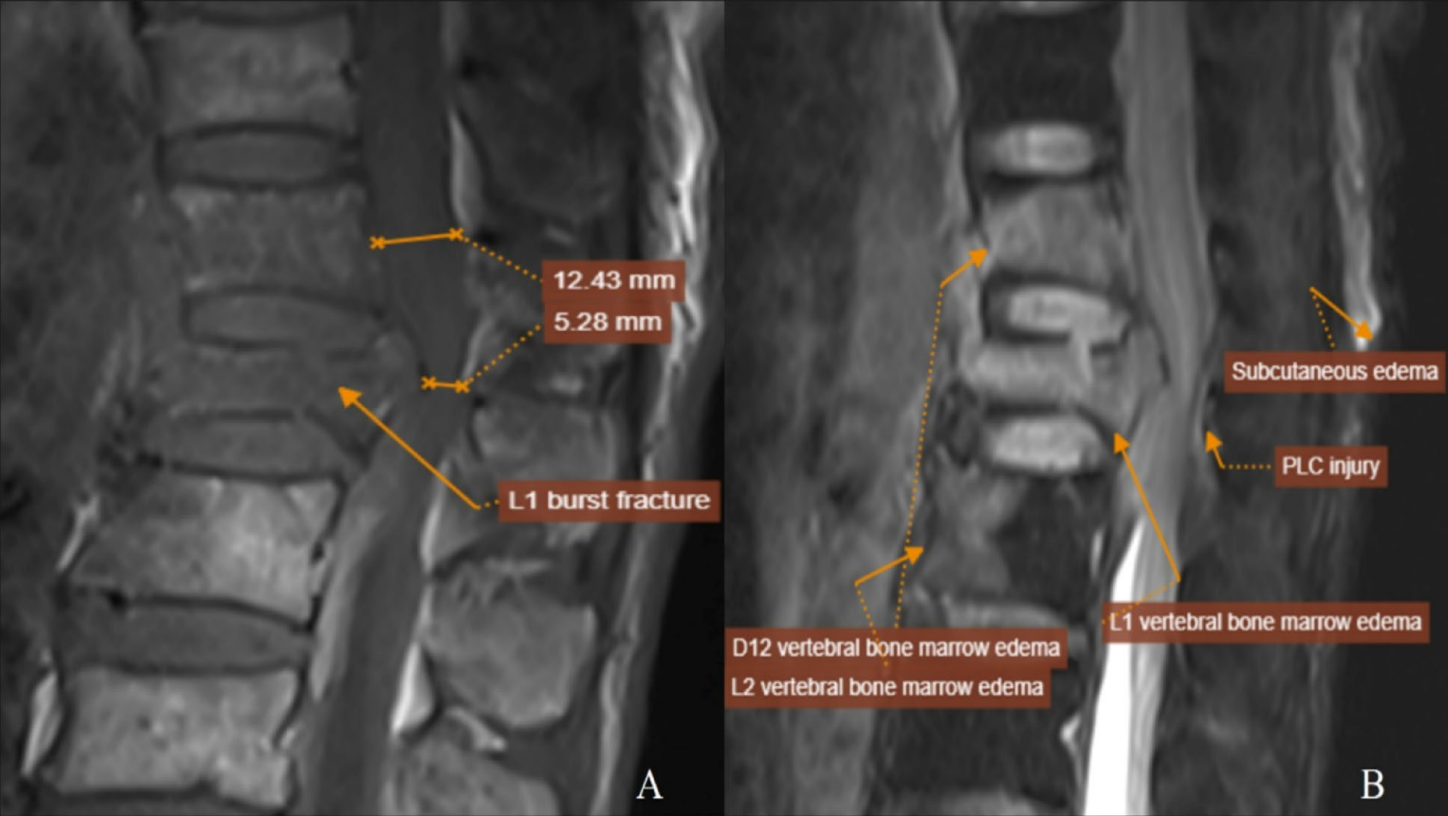
High-resolution MRI of another patient presenting with PLC disruption and severe canal compromise. MRI of L1 fracture with > 50% canal compromise (**A**) and PLC injury (**B**); mTLICS = 5 treated with posterior fixation (Images enhanced for contrast and clarity at 300 dpi resolution)

**The second case is shown in** Fig. 3

A male patient in his 60s was admitted following a high fall with trauma to the thoracolumbar junction. MRI demonstrated a burst fracture of the L1 vertebral body with > 50% spinal canal compromise (A) and high signal intensity in the posterior ligamentous complex (PLC), suggestive of ligamentous injury (B). The injury was scored as TL AOSIS = 4 (morphology: 5, PLC: 1), TLICS = 4 (morphology: 2, PLC: 2), and mTLICS = 5 (morphology: 3, PLC: 2). The patient underwent posterior internal fixation with pedicle screws.

#### Treatment strategies

Patients were stratified into three treatment categories based on multidisciplinary clinical decisions that incorporated neurological status, imaging findings, and institutional protocols in accordance with established guidelines (TLICS, TL AOSIS, WFNS Spine Committee).

Conservative management included orthotic bracing, pharmacologic pain control, and activity restriction. This approach was applied to neurologically intact patients (ASIA E) with stable fractures, typically involving less than 30% vertebral height loss, minimal canal compromise, and preserved PLC integrity on MRI. MIS, including percutaneous vertebroplasty or kyphoplasty, was undertaken within 7 days of injury. It was indicated for neurologically intact patients with radiologic signs of instability, such as ≥ 50% vertebral height loss, PLC disruption on STIR or Dixon sequences, or ≥ 50% canal compromise, in the absence of decompression criteria. Bone mineral density was not a limiting factor, aligning with current trends toward early image-guided stabilization in selected non-neurologic cases [11, 12]. Open surgery involved posterior instrumentation, with or without decompression, and was reserved for patients with neurological deterioration, marked canal compromise, or unstable burst fractures with PLC disruption. These procedures were also performed within 7 days, in accordance with institutional protocols.

The original mTLICS by Park supported binary treatment decisions without specifying MIS as a separate option. This study expanded the framework by formally recognizing MIS as an intermediate tier, aligning with evolving clinical practice. MIS thus served as a therapeutic bridge between conservative and open surgical management in neurologically intact patients requiring stabilization without decompression.

Treatment allocation was made independently of classification scores and was subsequently compared with TLICS, TL AOSIS, and mTLICS. In scenarios where existing systems offered ambiguous guidance, particularly TLICS scores of 3 to 4, the mTLICS was retrospectively evaluated for its ability to provide additional guidance based on MRI-derived indicators of instability [5, 6, 11, 16].

### Statistical analysis

All statistical analyses were conducted using SPSS version 22.0 (IBM Corp., USA) and MedCalc^®^ Statistical Software version 22.2 (MedCalc Software Ltd, Belgium). Continuous variables were summarized as means and standard deviations (SD) or medians and interquartile ranges (IQR), while categorical variables were presented as absolute counts and percentages.

Comparative analyses across the three treatment groups (conservative, MIS, and surgical) were performed using one-way ANOVA or Kruskal–Wallis tests for continuous variables, depending on the normality of distribution, and Chi-square or Fisher’s exact tests for categorical variables. We compared the predictive performance of three classification systems: TLICS, mTLICS, and the Thoracolumbar AO Spine Injury Score (TL AOSIS), a point-based extension of the AO Spine morphological classification that integrates neurologic status and clinical modifiers to guide treatment decisions. Receiver operating characteristic (ROC) curve analyses were conducted for each system to evaluate discriminatory capacity. The area under the curve (AUC) was reported, and optimal cutoff values were determined using Youden’s index, alongside sensitivity and specificity estimates.

To identify independent predictors of treatment modality, we applied multinomial logistic regression, treating treatment category as the dependent variable and using conservative management as the reference group. Independent variables included mTLICS, TLICS, TL AOSIS, and relevant clinical and radiological parameters. Given the unordered nature of the three treatment groups, this modeling approach was appropriate for capturing the structure of the outcome variable. Odds ratios (ORs) and 95% confidence intervals (CIs) were calculated for each covariate. Multicollinearity was assessed using variance inflation factor (VIF), and no variable exceeded a VIF threshold of 2.5. This approach allowed us to evaluate the independent statistical contribution of each classification system to treatment decision-making within a retrospective framework, without inferring causality.

Finally, decision curve analysis (DCA) was employed to assess the net clinical benefit of each classification system across a range of threshold probabilities (0.2–0.7). Particular emphasis was placed on the intermediate TLICS score range (3–4), which has been described as a clinical “gray zone” due to inconsistency in therapeutic guidance. A two-tailed p-value < 0.05 was considered statistically significant.

## Results

**Table 1:** Baseline demographic and clinical characteristics of the study population (*n* = 146)

Variable	Value
Age, mean ± SD (years)	57.3 ± 16.1
Sex, n (%)	
Male	78 (53.4%)
Female	68 (46.6%)
Occupation, n (%)	
Farmer	29 (19.9%)
Manual laborer / industrial worker	37 (25.3%)
Construction worker	16 (11.0%)
Retired	53 (36.3%)
Other occupations	11 (7.5%)
Mechanism of injury, n (%)	
Traffic accident	34 (23.3%)
Occupational accident	47 (32.2%)
Domestic fall / fall in daily activities	65 (44.5%)
Pain severity on admission (VAS)	Median: 5 (IQR: 2–7)
Neurological status (AIS classification)	
AIS A	2 (1.4%)
AIS C	6 (4.1%)
AIS D	18 (12.3%)
AIS E	120 (82.2%)
Fracture level, n (%)	
T11	8 (5.5%)
T12	35 (24%)
L1	62 (42.5%)
L2	24 (16.4%)
Other levels (T1–T10, L3–L5)	17 (11.6%)

*SD* standard deviation, *IQR* interquartile range, *VAS* visual analog scale, *AIS* American Spinal Injury Association Impairment Scale

The demographic and clinical characteristics of the study population are presented in Table 1. Among the 146 patients included in the study, male were slightly predominant (53.4%), with a mean age of 57.3 ± 16.2 years. Injuries most commonly occurred in retired individuals due to domestic falls, accounting for 36.3% and 44.5% of cases, respectively. The median pain score at admission was 5 (IQR: 2–7). Of note, 82.2% of patients were neurologically intact (AIS E) at initial presentation. The most frequently affected level was the L1 vertebral body, observed in 42.5% of cases

**Table 2:** Comparison of clinical, radiological, and classification features across treatment groups (*n* = 146)

Variable	Conservative (*n* = 50)	Minimally Invasive (*n* = 52)	Surgical (*n* = 44)	*p*-value
Age, mean ± SD (years)	54.7 ± 15.3	68,7 ± 11.8	46.9 ± 12.7	< 0.001^a^
Male sex, n (%)	26 (33.3%)	16 (20.5%)	36 (46.2%)	0.014^b^
VAS score, median (IQR)	2 (2–2)	5 (4–6)	7 (7–8)	< 0.001^c^
Fracture at L1, n (%)	17 (27.4%)	20 (32.3%)	25 (40.3%)	0.454^b^

AIS type	Conservative	Minimally Invasive	Surgical	*p*-value
AIS A	0 (0%)	0 (0%)	2 (100%)	—
AIS B	0 (0%)	0 (0%)	0 (0%)	—
AIS C	0 (0%)	0 (0%)	6 (100%)	—
AIS D	0 (0%)	1 (5.6%)	17 (94.4%)	< 0.001^b^
AIS E	49 (40.8%)	51 (42.5%)	20 (16.7%)	< 0.001^b^

Fracture type	Conservative	Minimally Invasive	Surgical	*p*-value
Wedge-compression	38 (71.7%)	15 (28.3%)	0 (0%)	0.002^b^
Burst fracture	11 (13.6%)	37 (45.7%)	33 (40.7%)	< 0.001^b^
Translation/rotation	0 (0%)	0 (0%)	3 (100%)	—
Distraction	0 (0%)	0 (0%)	9 (100%)	—

Degree of loss	Conservative	Minimally Invasive	Surgical	*p*-value
≥ 50%	1 (2.9%)	20 (57.1%)	14 (40%)	< 0.001^b^
< 50%	48 (43.3%)	32 (28.8%)	31 (27.9%)	0.085^b^

Degree	Conservative	Minimally Invasive	Surgical	*p*-value
≥ 50%	0 (0%)	0 (0%)	16 (100%)	—
< 50%	49 (37.7%)	52 (40%)	29 (22.3%)	0.027^b^

PLC score	Conservative	Minimally Invasive	Surgical	*p*-value
PLC = 0	44 (88%)	5 (10%)	1 (2%)	< 0.001^b^
PLC ≥ 1	5 (5.2%)	47 (49%)	44 (45.8%)	< 0.001^b^

Classification	Conservative	Minimally Invasive	Surgical	*p*-value
TLICS	1 (1–1.25)	4 (3–4)	6 (4–8)	< 0.001^c^
TL AOSIS	1 (1–5)	4 (2–4)	6 (4–9)	< 0.001^c^
mTLICS	1 (1–1.25)	3 (3–3)	6 (5–8)	< 0.001^c^

Neurological status (AIS), n (%)

Vertebral fracture morphology, n (%)

Vertebral body height loss, n (%)

Spinal canal compromise, n (%)

PLC injury (score), n (%)

Spinal classification scores (median, IQR)

* SD* standard deviation,* IQR* interquartile range,* VAS* visual analog scale, *PLC* posterior ligamentous complex,* ASIA* American Spinal Injury Association

^a^ ANOVA test

^b^Chi-square test

^c^Kruskal–Wallis test

Among 146 patients stratified into conservative (*n* = 50), minimally invasive (*n* = 52), and surgical (*n* = 44) treatment groups, statistically significant differences were observed across multiple clinical and imaging parameters, underscoring the heterogeneity of injury severity and its influence on treatment selection (Table 2).

**Age and sex** varied significantly across groups, with the highest mean age observed in the minimally invasive group (68.7 ± 11.8 years), followed by the conservative group (54.7 ± 15.3 years), and the youngest in the surgical group (46.9 ± 12.7 years) (*p* < 0.001, ANOVA). Although males were predominant overall, their proportion was highest in the surgical group (46.2%), compared to the conservative (33.3%) and minimally invasive (20.5%) groups (*p* = 0.014, Chi-square test), indicating gender-related variability in treatment patterns.

Pain severity, as measured by the Visual Analog Scale (VAS), exhibited a stepwise increase corresponding to the level of treatment invasiveness, with median scores of 2 (IQR: 2–2), 5 (IQR: 4–6), and 7 (IQR: 7–8) in the conservative, minimally invasive, and surgical groups, respectively (p < 0.001, Kruskal–Wallis test).

**Neurological status**, as classified by the ASIA Impairment Scale, also differed markedly. All patients in the surgical group presented with neurological deficits (94.4% ASIA C/D), whereas ASIA E (neurologically intact) was predominant in the conservative (40.8%) and minimally invasive (42.5%) groups (p < 0.001), highlighting the strong predictive value of neurological function in surgical decision-making.

Regarding **fracture morphology and location**, L1 was the most commonly affected level across all groups, without statistical significance (p = 0.454). However, fracture types varied considerably: wedge-compression fractures were predominant in the conservative group (71.7%), while burst fractures were more frequent in the minimally invasive (45.7%) and surgical (40.7%) groups. Notably, translation/rotation and distraction injuries were exclusively observed in the surgical group, reflecting more severe mechanical instability (p < 0.001).

On **MRI-based radiological features**, the proportion of patients with ≥50% vertebral height loss was highest in the minimally invasive group (57.1%), followed by the surgical (40.0%) and conservative groups (2.9%) (p < 0.001). Spinal canal compromise ≥50% was recorded exclusively in the surgical group (100%) (p < 0.001). Similarly, PLC injury (score ≥1) was observed in 97.7% of surgical cases and 90.4% of minimally invasive cases, but only in 12% of conservative cases (p < 0.001), indicating the critical role of MRI in detecting instability and guiding escalation of care.

The **classification scores** from all three systems, namely TLICS, TL AOSIS, and mTLICS, showed progressive increases consistent with treatment invasiveness. Specifically, median TLICS scores were 1 (IQR: 1–1.25), 4 (IQR: 3–4), and 6 (IQR: 4–8) across conservative, minimally invasive, and surgical groups, respectively. AO scores followed a similar pattern: 1 (IQR: 1–5), 4 (IQR: 2–4), and 6 (IQR: 4–9), while mTLICS yielded 1 (IQR: 1–1.25), 3 (IQR: 3–3), and 6 (IQR: 5–8), respectively. All comparisons were statistically significant (p < 0.001, Kruskal–Wallis test).

Taken together, these findings underscore the capacity of mTLICS to facilitate nuanced treatment stratification in thoracolumbar trauma patients across treatment pathways and further support the clinical utility of the mTLICS system in aligning classification scores with real-world treatment decisions, particularly in the context of ambiguous or intermediate injury severity.

**Table 3:** Multinomial logistic regression identifying predictors of treatment modality (reference group: conservative)

Predictor variable	Treatment group		Odds Ratio (OR)	95% Confidence Interval (CI)	*p*-value
Age	Minimally invasive		1.074	0.991–1.164	0.083
	Surgical		0.909	0.753–1.098	0.321
VAS score	Minimally invasive		1.530	0.726–3.224	0.264
	Surgical		1.548	0.226 – 10.595	0.656
TLICS score	Minimally invasive		0.004	5.954e⁻⁷ – 30.270	0.228
	Surgical		0.005	6.705e⁻⁷ – 38,045	0.246
TL AOSIS	Minimally invasive		0.201	0.01–3.868	0.288
	Surgical		0.106	0.004–3.019	0.189
mTLICS score	Minimally invasive		31.219	2.32–420.086	**0.009**
	Surgical		1338.367	15.685–1141986.615	**0.002**
Neurologically intact	Minimally invasive		0	0 – b	1.000
	Surgical		0.032	0.032–0.032	–
Burst fractures	Minimally invasive		4.393e⁻⁷	4.393e⁻⁷ – 4.393e⁻⁷	–
	Surgical		5.533e⁻²⁷	0 – b	0.996
PLC injury (grade ≥ 1)	Minimally invasive		195542.645	0.010–3.703e¹²	0.154
	Surgical		4855875.647	0 – b	0.994
Spinal canal compromise ≥ 50%	Minimally invasive		0	0–0	–
	Surgical		6763.266	0 – b	0.999

* OR* Odds Ratio,* CI* Confidence Interval (95%)

“b”Confidence interval not computable due to complete or quasi-complete separation (extremely sparse data). “—”: Model could not estimate effect due to limited cases or perfect prediction

Bold values indicate statistical significance at *p* < 0.05

The multinomial logistic regression model demonstrated excellent overall fit (Chi-square = 269.999; df = 22; *p* < 0.001), with a Nagelkerke R^2^ of 0.948, indicating that the model explained approximately 94.8% of the variance in treatment allocation across the three groups (conservative, minimally invasive, and surgical).

The detailed results of the multinomial logistic regression analysis are presented in Table 3. Among all analyzed variables, the mTLICS classification emerged as the strongest independent predictor, showing statistically significant associations with both minimally invasive intervention (OR = 31.2; 95% CI: 2.32–420.09; *p* = 0.009) and surgical treatment (OR = 1,338.4; 95% CI: 15.69–1,141,986.2; *p* = 0.002). The Likelihood Ratio Test further confirmed that mTLICS was the only variable that significantly contributed to the predictive model (Chi-square = 21.114; df = 2; *p* < 0.001), whereas the traditional classifications TLICS (*p* = 0.179) and TL AOSIS (*p* = 0.464) failed to demonstrate statistical significance. These findings strongly support the superior discriminative performance of mTLICS in stratifying treatment decisions for thoracolumbar spine injuries, particularly when distinguishing among the three-tiered management strategies.

Integrating quantitative MRI-derived parameters into mTLICS, such as PLC injury grading, vertebral height loss, and spinal canal compromise, likely accounts for its enhanced predictive accuracy and clinical relevance, especially in guiding individualized treatment decisions in real-world practice.

Diagnostic Accuracy of Classification Systems: The diagnostic performance of the three classification systems, namely mTLICS, TLICS, and TL AOSIS, was evaluated using receiver operating characteristic (ROC) curve analysis across both the full study cohort and the intermediate-score subgroup (TLICS = 3–4).

**Fig. 4:**
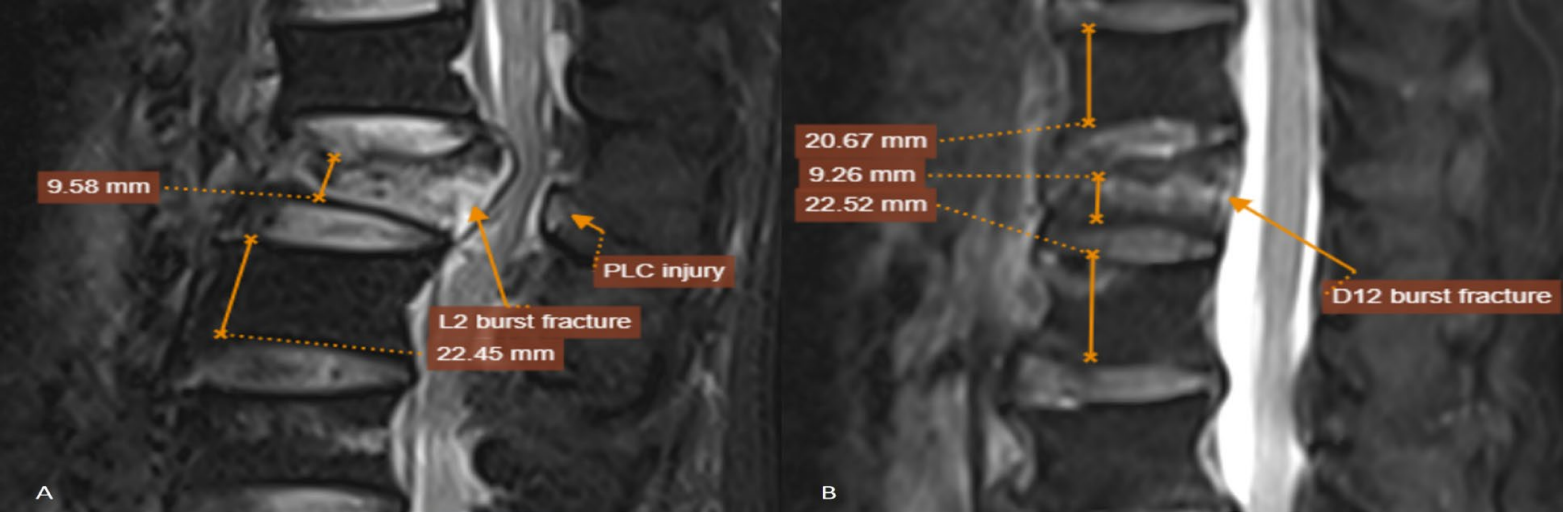
**A** A 54-year-old male presented after a 3-meter fall with an L2 burst fracture, > 50% vertebral height loss, and posterior ligamentous complex (PLC) edema without definite disruption. The mTLICS score was 5, and the patient underwent posterior spinal fixation. **B** A 63-year-old female presented after an indoor fall with a D12 burst fracture showing > 50% vertebral height loss without PLC injury (mTLICS = 3). The patient was treated with percutaneous vertebroplasty

Full Cohort Analysis: The diagnostic accuracy of each classification system across the full patient cohort is presented in Table 4. As illustrated in Fig. 4, mTLICS consistently demonstrated superior discriminative accuracy compared to TLICS and TL AOSIS classifications in predicting treatment allocation. The area under the curve (AUC) for mTLICS ranged from 0.94 to 1.00 across all three treatment comparisons: conservative versus minimally invasive, conservative versus surgical, and minimally invasive versus surgical. Sensitivity and specificity for mTLICS were 95.6–100% and 87.8–98%, respectively. Notably, the AUC for distinguishing conservative versus surgical treatment reached the maximum value of 1.000 (95% CI: 0.961–1.000; *p* < 0.001). In contrast, the TLICS and TL AOSIS systems yielded lower AUCs of 0.978 and 0.983, respectively, with reduced classification precision.

**Table 4:** ROC performance of the three classification systems in the entire patient cohort (*n* = 146)

Classification System	Treatment Comparison	AUC (95% CI)	*p*-value	Cut-off Score	Sensitivity (%)	Specificity (%)
TL AOSIS	Conservative vs. Intervention	0.887 (0.808–0.941)	< 0.001	> 1	100	75.6
	Conservative vs. Surgery	0.983 (0.931–0.999)	< 0.001	> 3	97.8	89.8
	Intervention vs. Surgery	0.887 (0.807–0.942)	< 0.001	> 4	71.1	90.4
TLICS	Conservative vs. Intervention	0.913 (0.84–0.96)	< 0.001	> 1	100	77.6
	Conservative vs. Surgery	0.978 (0.924–0.997)	< 0.001	> 3	97.8	89.8
	Intervention vs. Surgery	0.873 (0.79–0.932)	< 0.001	> 4	62.2	100
mTLICS	Conservative vs. Intervention	0.94 (0.874–0.977)	< 0.001	> 2	98.1	87.8
	Conservative vs. Surgery	1 (0.961–1)	< 0.001	> 3	100	98
	Intervention vs. Surgery	0.987 (0.94–0.999)	< 0.001	> 4	95.6%	96.2

*AUC* Area Under the ROC Curve, *CI* Confidence Interval (95%), *Cut-off*  Optimal threshold based on Youden index

Intermediate score subgroup (TLICS = 3–4): To assess model performance in scenarios with greater therapeutic uncertainty, a separate ROC analysis was performed in the subgroup of patients with TLICS scores between 3 and 4. The corresponding findings are presented in Table 5. mTLICS retained outstanding discriminative capacity in this subset, achieving an AUC of 0.991 for conservative versus surgical and 0.965 for minimally invasive versus surgical comparisons, with both sensitivity and specificity exceeding 87%. In contrast, the diagnostic accuracy of the TLICS and TL AOSIS systems was substantially lower in this intermediate-score subgroup. AUC values for both models consistently fell below 0.76, and corresponding sensitivity and specificity frequently dropped below 33%, highlighting their reduced clinical utility in guiding treatment decisions under conditions of radiological or neurological ambiguity.

**Table 5:** ROC performance of the three classification systems in the intermediate score group (TLICS = 3–4)

Classification System	Treatment Comparison	AUC (95% CI)	*p*-value	Cut-off Score	Sensitivity (%)	Specificity (%)
TL AOSIS	Conservative vs. Intervention	0.55 (0.407–0.687)	0,5	≤ 2	32.6	100
	Conservative vs. Surgery	0.754 (0.532–0.908)	0.001	> 4	31.3	100
	Intervention vs. Surgery	0.726 (0.597–0.831)	< 0.001	> 3	100	37
TLICS	Conservative vs. Intervention	0.52 (0.379–0.659)	0.838	≤ 3	32.6	71.3
	Conservative vs. Surgery	0.643 (0.418–0.829)	0.121	> 3	100	28.6
	Intervention vs. Surgery	0.663 (0.532–0.778)	< 0.001	> 3	100	32.6
mTLICS	Conservative vs. Intervention	0.596 (0.453–0.729)	0.331	> 2	100	14.3
	Conservative vs. Surgery	0.991 (0.836–1)	< 0.001	> 4	87.5	100
	Intervention vs. Surgery	0.965 (0.884–0.995)	< 0.001	> 4	87.5	95.7

*AUC* Area Under the ROC Curve, *CI*  Confidence Interval (95%). *Cut-off*  Optimal threshold based on Youden index

The comparative ROC performance across classification systems is visualized in Fig. 4, further emphasizing the superior diagnostic capability of mTLICS across treatment strata and score subgroups.

These findings reinforce the clinical utility of mTLICS as a high-fidelity, MRI-enhanced classification framework for treatment stratification in thoracolumbar injuries. Its superior diagnostic accuracy is attributable to the systematic integration of quantitative imaging markers, specifically posterior ligamentous complex (PLC) disruption, ≥ 50% vertebral body height loss, and ≥ 50% spinal canal compromise. By translating radiological instability into objective scoring components, mTLICS effectively bridges the gap between morphological assessment and therapeutic decision-making, particularly in intermediate-score scenarios where conventional systems often fail to provide definitive guidance.

**Fig. 5:**
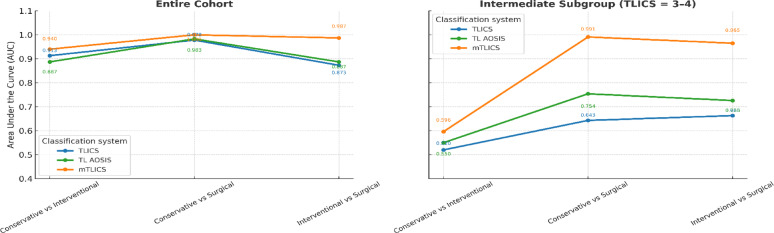
Comparative diagnostic performance (AUC) of mTLICS, TL AOSIS, and TLICS classification systems in stratifying thoracolumbar injuries. The left panel shows ROC results in the full cohort, while the right panel focuses on the intermediate score subgroup (TLICS = 3–4)

Figure 5 illustrates the distribution of mTLICS scores across three treatment groups. Patients managed conservatively had the lowest scores (median = 1), with all cases below the intervention threshold (mTLICS < 4). The minimally invasive group showed a tight clustering at score 4, corresponding to the proposed intermediate zone. Surgical patients exhibited the highest scores (median = 6), with most exceeding the surgical threshold (mTLICS ≥ 5).

 This stratified pattern supports the discriminative ability of mTLICS in guiding three-tiered treatment decisions. Notably, the score of 4 clearly delineates candidates for minimally invasive procedures, which represents an area of ambiguity in traditional classification systems.

**Fig. 6:**
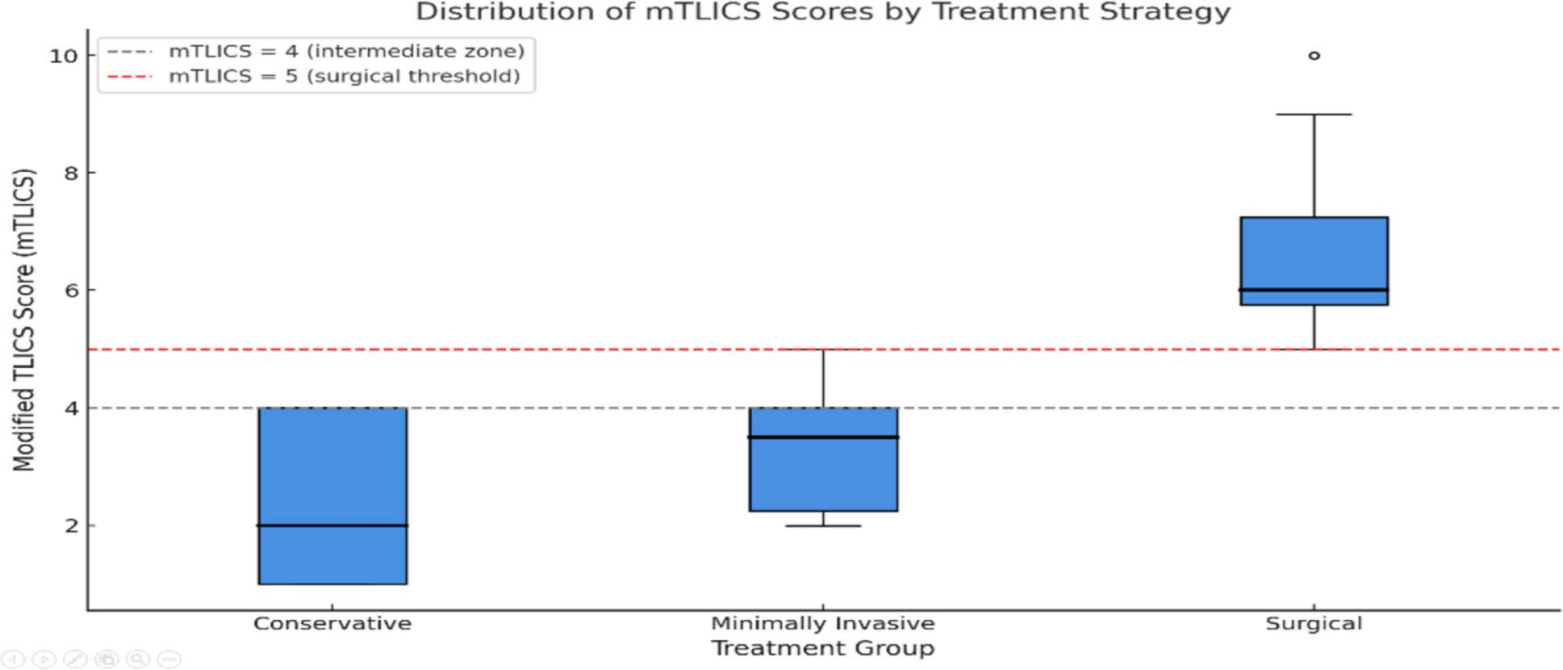
Distribution of mTLICS scores by treatment strategy

**Fig. 7:**
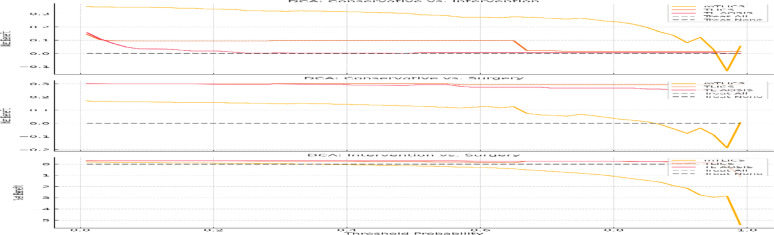
DCA comparing the net clinical benefit of mTLICS, TLICS, and TL AOSIS in three treatment comparisons: (top) conservative vs. intervention, (middle) conservative vs. surgery, and (bottom) intervention vs. surgery. mTLICS consistently demonstrated the highest net benefit across threshold probabilities, particularly within the 0.3–0.6 range, where therapeutic decision-making is most uncertain

 DCA was performed across three pairwise treatment comparisons to assess the net clinical benefit of each classification system. As illustrated in Fig. 6, the mTLICS consistently yielded the highest net clinical benefit across all threshold probabilities (0.2–0.7), with a marked advantage within the intermediate range of 0.3–0.6, where therapeutic decision-making is often most uncertain (see Fig. 7).

 In all comparisons, including conservative versus intervention, conservative versus surgery, and intervention versus surgery, mTLICS outperformed both TLICS and TL AOSIS. The net benefit curves for TLICS and TL AOSIS frequently fell below the “treat-all” and “treat-none” reference lines, particularly in the 0.3–0.6 probability zone, indicating their limited utility in resolving clinical ambiguity.

 These findings further validate the role of mTLICS as a superior, image-informed stratification tool, especially in neurologically intact patients with MRI-based indicators of mechanical instability.

##  Discussion

 This study underscores the superior clinical utility of mTLICS in stratifying thoracolumbar injuries into three therapeutic categories: conservative, MIS, and surgical. Compared to TLICS and TL AOSIS, mTLICS demonstrated enhanced discriminative capacity, particularly within the intermediate score range (TLICS = 3–4), which has long posed challenges in treatment decision-making. Although originally developed to guide binary choices, the mTLICS framework did not account for MIS. Our study expands this model by defining MIS as an intermediate tier for neurologically intact patients (ASIA E) with MRI-detected instability who do not require decompression. This refinement aligns with evolving clinical practice and enables a more nuanced evaluation of classification performance, particularly in the TLICS 3–4 “gray zone”.

 mTLICS and the Intermediate Score Gap:

 In both the full cohort (*n* = 146) and the TLICS 3–4 subgroup, mTLICS consistently outperformed its predecessors. The system achieved AUCs ranging from 0.94 to 1.00 across all treatment comparisons, with the highest value (AUC = 1.000) observed for conservative vs. surgical stratification. In the TLICS 3–4 subgroup, mTLICS maintained excellent discriminatory accuracy, with an AUC of 0.991 for conservative vs. surgical and 0.965 for minimally invasive vs. surgical, while TLICS and TL AOSIS underperformed significantly (AUC < 0.76, sensitivity/specificity < 33%).

 These results underscore the clinical value of incorporating quantitative MRI-derived criteria, such as graded posterior ligamentous complex (PLC) injury, ≥ 50% vertebral height loss, and ≥ 50% spinal canal stenosis, into treatment decision-making. Conventional systems like TLICS and TL AOSIS lack this level of imaging-based detail and provide limited guidance in cases of diagnostic ambiguity [3, 4]. In our cohort, 90.4% of patients who underwent minimally invasive procedures had PLC scores ≥ 1 and were neurologically intact (AIS E), reinforcing the relevance of MRI-based instability markers in stratifying treatment strategies.

Comparative Evidence and Model Validity:

This study builds upon the foundational work of Park [5] and Withrow [6], who developed and validated earlier versions of the mTLICS system but applied it exclusively to dichotomous treatment pathways (i.e., conservative versus surgical). To our knowledge, this is among the first studies to implement and validate a structured three-tier stratification model that includes minimally invasive interventions and is based on real-world clinical data. Consistent with An et al., who reported that TL AOSIS offers marginal advantage over TLICS in certain cases, our findings reaffirm that both conventional systems underperform in stratifying intermediate-score injuries, thereby reinforcing the added clinical value of mTLICS. Within this model, mTLICS emerged as the strongest independent predictor of treatment allocation (OR > 1300 for surgical intervention; *p* < 0.001), whereas TLICS and TL AOSIS systems failed to demonstrate statistical significance. These results were further supported by excellent model performance (Nagelkerke R² = 0.948).

Among the 24 studies reviewed in Table 6, only Park and Withrow directly evaluated mTLICS in clinical cohorts [5, 6]. In contrast, the majority of remaining studies underscore its necessity by highlighting key limitations of current systems. Notably, Khil, Li, Duramaz, and Smith et al. have reported significant inconsistencies in patients with TLICS = 4—including conservative treatment failure rates of up to 32% and frequent deviations from score-based recommendations [13, 14, 17, 18]. Furthermore, Lucasti [13] and Gonzales-Portillo [18] documented that up to 28% of real-world treatment decisions were discordant with TLICS-based guidance, reinforcing the need for MRI-informed classification frameworks that better align with clinical outcomes.

**Table 6:** Summary of studies supporting mTLICS classification development (Sorted by Year)

Author	Year	Study Type	Main Academic Contribution	Relevance to mTLICS
Vaccaro et al. [3]	2005	Original classification	Developed TLICS with 3-point PLC scale; ambiguity at score = 4	Foundation requiring refinement with imaging
Joaquim & Patel [2]	2013	Surgical decision-making review	Emphasized role of imaging in detecting instability	Advocates MRI for modern classification
Kepler et al. [4]	2016	Morphology-based classification	Created AO Spine system without quantitative PLC assessment	Inadequate for individualized decisions
Kumar et al. [8]	2016	Pictorial MRI review	Highlighted MRI utility in detecting occult fractures, PLC injury	Reinforces centrality of MRI in classification
Pneumaticos et al. [19]	2016	Retrospective TLICS analysis	Suggested TLICS = 4 can be treated conservatively if AIS E	Supports need for stratified, MRI-informed system
An et al. [7]	2020	Comparative classification study	Compared TL AOSIS and TLICS in treatment decision-making; found only marginal advantage of TL AOSIS	Reinforces that both conventional systems are limited in guiding treatment, especially in borderline cases; supports need for MRI-based enhancement as in mTLICS
Park et al. [5]	2016	Classification proposal	Proposed mTLICS with MRI-based modifiers (height loss, canal compromise)	Core development of mTLICS
Su et al. [16]	2020	Theoretical model refinement	Critiqued three-column model lacking imaging data	Conceptual support for mTLICS
Duramaz et al. [17]	2020	Treatment outcome study	Found no benefit of surgery in TLICS = 4 cases	Suggests tailored treatment based on instability
Park et al. [20]	2020	Validation study	TLICS valid with deficits; poor for AIS E patients	Justifies mTLICS for neurologically intact cases
Mehta et al. [8]	2021	Diagnostic accuracy study	MRI sensitivity: 95%; specificity: 92.5% for PLC injury	MRI is the gold standard
WFNS [21]	2021	Global guidelines	Recommends MRI for instability with unclear neurology	Supports MRI-based decision-making
Smith et al. [14]	2021	Outcome-based analysis	TLICS did not predict outcomes reliably	Argues for refined systems like mTLICS
Laurita et al. [22]	2022	Imaging evaluation	Upright X-rays missed instability	Confirms MRI superiority
Nagi & Sakr [23]	2022	MRI diagnostic comparison	Found TLICS accuracy improved with MRI	Endorses imaging integration
Gonzales-Portillo [18]	2023	Real-world TLICS application	28% mismatch between TLICS score and treatment	Real-world justification for mTLICS
Lucasti et al. [13]	2023	Observational study	Surgery used even with TLICS < 4	MRI may rationalize surgical decisions
Song et al. [12]	2023	Surgical outcome comparison	Percutaneous = open surgery for A3 fractures	Supports minimally invasive option for score = 4
Aly et al. [10]	2023	Academic proposal	Proposed standardized MRI-based PLC scoring	Supports structural refinement of TLICS
Li et al. [24]	2023	Meta-analysis	TLICS = 4: equivalent outcomes in surgery and conservative	Encourages stratification via mTLICS
Gao et al. [11]	2024	Umbrella review protocol	Evaluated vertebroplasty/kyphoplasty in stable fractures	Supports intermediate-tier treatment stratification
Blixt et al. [25]	2024	Registry-based reliability study	Low interobserver agreement for TLICS, AO	mTLICS improves reproducibility
Khil et al. [15]	2024	Treatment failure analysis	32% failed conservative care with TLICS 4–5	MRI-based stratification is essential
Withrow et al. [6]	2025	Validation study	mTLICS: AUC = 0.96; *r* = 0.77 with treatment	Validates mTLICS accuracy and reliability

Imaging-Based Individualization and Clinical Utility:

Numerous studies have affirmed the pivotal role of MRI in the evaluation of thoracolumbar spine injury. Mehta and Nagi & Sakr reported that MRI achieves over 95% sensitivity in detecting PLC injuries, far exceeding the diagnostic yield of conventional imaging modalities [8, 23]. Joaquim & Patel further underscored its importance in identifying occult instability, particularly in patients without neurological deficits [2]. Despite this, widely used classification systems such as TLICS and TL AOSIS continue to rely primarily on subjective or morphology-based criteria. The mTLICS system directly addresses this gap by integrating standardized, quantitative MRI parameters into its scoring framework, thereby enhancing objectivity and clinical precision.

Our DCA demonstrated the clinical advantage of mTLICS across the entire range of threshold probabilities (0.2–0.7), consistently yielding the highest net benefit. Most notably, it offered clear decision-making utility within the 0.3–0.6 range—precisely where clinical ambiguity is most pronounced and where TLICS and TL AOSIS frequently fell below the “Treat-All” and “Treat-None” reference lines. These findings support recent recommendations by Gao, Park, and Su to adopt individualized, imaging-guided classification models tailored to modern treatment paradigms [5, 11, 16].

Additionally, studies from both high- and low-resource settings have revealed considerable variability in treatment decisions for thoracolumbar injuries, often due to limited MRI access and lack of standardized image interpretation. Almigdad [26] and Shu [27] noted conservative treatment bias in settings with restricted imaging resources, while Blixt [25] reported low interobserver reliability with traditional classifications. By providing explicit MRI-based thresholds, such as a score of 4 for minimally invasive intervention, mTLICS bridges theoretical classification with clinical reality, particularly in AIS E patients, who accounted for over 80% of our cohort.

### Limitations and future directions

This study has several limitations. First, its retrospective, single-center design may limit external validity and generalizability. Prospective, multicenter studies are warranted to confirm these findings across broader clinical settings. Second, although model performance was assessed using ROC and DCA, complementary metrics such as the Net Reclassification Index (NRI) and Integrated Discrimination Improvement (IDI) were not included, which could provide additional insights into the incremental predictive value of mTLICS over existing systems. Third, sparse data bias was observed in certain regression models, as reflected by wide confidence intervals and inflated odds ratios; therefore, future analyses may benefit from regularization approaches such as Firth’s penalized likelihood or Bayesian shrinkage to improve model stability in datasets with limited events per variable. Fourth, all MRI assessments were performed by a single radiologist, and interobserver agreement was not evaluated. Incorporating multi-reader evaluations and reporting interrater reliability would strengthen reproducibility and external applicability. Fifth, long-term functional outcomes, such as Visual Analog Scale (VAS) and Oswestry Disability Index (ODI) scores, were not available, which limits the ability to assess patient-reported functional recovery. Sixth, the study cohort included both high-energy trauma in younger adults and low-energy osteoporotic fractures in older patients, introducing etiological heterogeneity; as a result, stratified analyses are needed to clarify comparative performance across these subgroups. Seventh, polytrauma patients were excluded to ensure timely MRI acquisition and reduce confounding, which may have biased the sample toward isolated, lower-energy injuries. Eighth, only patients who underwent MRI were included, introducing potential selection bias. This reflects institutional practice favoring MRI in neurologically intact patients or those with inconclusive CT findings, thereby limiting applicability in settings where MRI is unavailable or not routinely performed. Finally, although clinicians at our institution were familiar with the mTLICS framework through prior academic exposure, all treatment decisions during the study period adhered to multidisciplinary consensus in line with institutional protocols and established guidelines (TLICS, TL AOSIS, WFNS Spine Committee). The mTLICS scores were calculated retrospectively from anonymized imaging and clinical data after treatment completion and were not used prospectively to guide patient management. Nevertheless, potential incorporation bias cannot be fully excluded, underscoring the necessity for prospective, blinded validation in independent cohorts.

## Conclusions 1

This study shows that mTLICS offers superior accuracy in stratifying thoracolumbar spine injuries into three treatment tiers: conservative, MIS, and surgical. It outperformed TLICS and TL AOSIS, particularly in the intermediate-score subgroup (TLICS = 3–4), with AUC values ranging from 0.94 to 1.00. Multinomial logistic regression identified mTLICS as the only system independently associated with treatment selection. This likely reflects its integration of MRI-based indicators, including posterior ligamentous complex integrity, vertebral height loss, and canal compromise, which enable a more precise evaluation of instability. These results highlight the clinical value of mTLICS, especially in neurologically intact patients with MRI-detected instability. However, as clinicians at the study site were familiar with the mTLICS framework through prior academic exposure, potential incorporation bias cannot be fully excluded. The mTLICS scores were calculated retrospectively after treatment completion and were not used prospectively to determine patient management. Additionally, the retrospective design precludes causal inference, underscoring the need for prospective, multicenter studies with long-term outcomes to confirm its generalizability and clinical relevance in contemporary spine trauma care.

## Data Availability

All data generated or analysed during this study are included in this published article.

## Abbreviations

TLICS: Thoracolumbar Injury Classification and Severity Score
TL AOSIS: the Thoracolumbar AOSpine Injury Score
mTLICS: the modified TLICS (Modified thoracolumbar injury classification and severity score)
PLC: Posterior Ligamentous Complex
MRI: Magnetic Resonance Imaging
MIS: Minimally Invasive Surgery
ASIA: American Spinal Injury Association Scale
VAS: Visual Analog Scale
ROC: Receiver Operating Characteristic curve
AUC: Area Under the Receiver Operating Characteristic Curve
DCA: Decision Curve Analysis
